# Standard of care in advanced HIV disease: review of HIV treatment guidelines in sub-Saharan African countries—an extension study of eight countries

**DOI:** 10.1186/s12981-025-00733-9

**Published:** 2025-03-29

**Authors:** Thomas C. Scheier, Tafese Beyene Tufa, Torsten Feldt, Yasmine Hardy, Albert Minga, Raoul Moh, Albertino Damasceno, Lucia Chambal, Francine Ntoumi, Carine Kades, Leopold Bitunguhari, Osee R. Sebatunzi, Marco Missanga, Katanekwa Njekwa, Monde Muyoyeta, Sumathy Rangarajan, Graeme Meintjes, Dominik Mertz, John W. Eikelboom, Sean Wasserman

**Affiliations:** 1 Population Health Research Institute, McMaster University and Hamilton Health Sciences, Hamilton, ON Canada; 2 Asella Referral and Teaching Hospital, College of Health Sciences, Arsi University, P.O. Box 04, Asella, Ethiopia; 3 Hirsch Institute of Tropical Medicine (HITM), Heinrich-Heine University, P.O. Box 04, Asella, Ethiopia; 4 Department of Gastroenterology, Hepatology and Infectious Diseases, University Hospital of Düsseldorf (UKD), 40225 Düsseldorf, Germany; 5 Directorate of Medicine, Komfo Anokye Teaching Hospital, Kumasi, Ghana; 6 Centre Médical de Suivi des Donneurs de Sang (CMSDS-CNTSCI), Abidjan, Côte d’Ivoire; 7 Programme PAC-CI, Site ANRS de Côte d’Ivoire, Abidjan, Côte d’Ivoire; 8 Unité Pédagogique de Dermatologie et Infectiologie, Université Félix Houphouët-Boigny, Abidjan, Côte d’Ivoire; 9 Faculty of Medicine, Eduardo Mondlane University, Maputo, Mozambique; 10 Maputo Central Hospital, Maputo, Mozambique; 11 Fondation Congolaise pour la Recherche Médicale, Brazzaville, Republic of the Congo; 12 Institute for Tropical Medicine, University of Tübingen, Tübingen, Germany; 13 Department of Internal Medicine, University Teaching Hospital of Kigali, Kigali, Rwanda; 14 Department of Internal Medicine, School of Medicine and Pharmacy, University of Rwanda, Kigali, Rwanda; 15 NIMR-Mbeya Medical Research Center, Mbeya, Tanzania; 16 Center for Infectious Disease Research in Zambia (CIDRZ), P.O. Box 34681, 10101 Lusaka, Zambia; 17 Tuberculosis Programs-Director, Centre for Infectious Disease Research, P.O. Box 34681, 10101 Lusaka, Zambia; 18 Wellcome Discovery Research Platforms in Infection, Centre for Infectious Diseases Research in Africa, Institute of Infectious Disease and Molecular Medicine, University of Cape Town, Observatory, Cape Town, Republic of South Africa; 19 Department of Medicine, University of Cape Town, Cape Town, South Africa; 20 Blizard Institute, Queen Mary University of London, London, UK; 21 Division of Infectious Diseases, Department of Medicine, McMaster University, Hamilton, ON Canada; 22 Department of Health Research Methodology, Evidence, and Impact, Faculty of Health Sciences, McMaster University, Hamilton, ON Canada; 23 Institute for Infection and Immunity, City St George’s, University of London, London, UK

**Keywords:** Advanced HIV disease, Guidelines, Sub-Saharan Africa, Antiretroviral therapy

## Abstract

**Introduction:**

The World Health Organization (WHO) has published guidelines for the management of patients with advanced HIV disease (AHD) but mortality remains high. Adoption of WHO recommendations by national guidelines is poorly documented. We aimed to extend our prior review of six national management guidelines by including additional countries from sub-Saharan Africa.

**Methods:**

We identified guidelines of eight additional countries participating in a multicountry trial of azithromycin prophylaxis for AHD. Data was extracted in five domains including definition of AHD (1 item), screening (6 items), prophylaxis (6 items), supportive care (1 items), and HIV treatment (4 items) and scored agreement of each national guideline with the WHO guidelines.

**Results:**

Six of the eight national guidelines had a designated section for AHD. Compared with the WHO guideline, the agreement score for national guidelines was between 7 and 17 out of 18, whereby disagreement is mainly driven by missing information. None of the national guidelines had more than three items not in agreement with the WHO guidelines, and the maximum number of items not addressed by any one guideline was eight. Main areas of disagreement were the targeted population for start of ART in presence of tuberculosis meningitis (1/8 in agreement) and urine lipoarabinomannan screening (2/8 in agreement). The targeted population group for cotrimoxazole prophylaxis and its discontinuation was in line with the WHO recommendations in 3/8 national guidelines. Except one guideline, all documents showed similar overall agreement, irrespectively of publication date.

**Conclusion:**

National guidelines for the management of people with AHD are broadly in agreement with WHO guidelines. Main areas of disagreement are recommendations regarding urine lipoarabinomannan screening, cotrimoxazole prophylaxis and start of antiretroviral therapy in presence of tuberculosis.

## Introduction

Advanced HIV disease (AHD), defined as a CD4 count of < 200 CD4 + cells/mm^3^ or World Health Organization (WHO) clinical stage 3 or 4, affects about 4 million adults globally [1–3]. People with AHD are at high risk of death in the first months after entering or re-entering care, with progressively higher mortality in those with lower CD4 counts [4–6]. Even in the setting of a contemporary randomized controlled trial investigating enhanced prophylactic treatments, more than 10% of participants died within six months [7]. The cause of death is often unknown, but opportunistic and bacterial infections are likely key drivers [8].

Guidelines for the management of people with AHD were first published by the WHO in 2017 [2]. These guidelines provide evidence-based recommendations, including diagnosis, prophylaxis and pre-emptive treatment, and adherence support. Subsequent updates have incorporated emerging evidence and highlighted specific aspects such as care of people with AHD who are seriously ill and the diagnosis of opportunistic infections [9, 10].

We have previously reported on the uptake of the WHO guideline recommendations in national guidelines of six countries in sub-Saharan Africa who participated in the vanguard phase of the *Reducing Mortality In Adults With Advanced HIV Disease* trial (REVIVE trial, NCT05580666) [11]. Since our previous publication, the REVIVE trial has expanded to include an additional eight countries. Here, we evaluate the uptake by national guidelines from these eight countries of the latest WHO guideline AHD recommendations as summarized in the 2021 document “*Consolidated guidelines on HIV prevention, testing, treatment, service delivery and monitoring: recommendations for a public health approach”* [12].

## Methods

The methodology for this review was identical to the previously published report and described in detail there [11]. In brief, lead investigators of participating countries provided national guideline documents outlining the management of people with AHD. Data for the following categories were extracted from national guidelines and the WHO 2021 document:

1. Definition of AHD (1 item)
2. Screening (6 items)
3. Prophylaxis (6 items)
4. Supportive care (1 item)
5. HIV treatment (4 items)

Extracted data was presented to the national investigator to review and validate. Disagreements regarding the accuracy of the extracted data were resolved by discussion.

National guideline recommendations were compared with those in the WHO 2021 document and scored as follows: Not addressed (0 points): no information for the respective item was found within the national guidelines. No agreement (also 0 points): national recommendation not in line with the WHO 2021 guidelines. Agreement (1 point): extracted data for an item in the national guideline matched the WHO 2021 guideline recommendations. Partial agreement (0.5 points): data of the national guideline included similar but not identical criteria (e.g., WHO 2021: CD4 +  < 100 cells/mm^3^, National guideline: CD4 +  < 200 cells/mm^3^). Scores were calculated for each national guideline and presented as bar graphs. Overall agreement was presented based on the publication year of the guideline.

## Results

We reviewed national guidelines from the following eight countries: Ethiopia, Ghana, Ivory Coast, Mozambique, Republic of Congo, Rwanda, Tanzania, and Zambia [13–20]. Six had a specific section for AHD. Guidelines were published between 2019 and 2023.

Information extracted from national guidelines is presented in Table 1 and agreement with the WHO 2021 document in Table 2. The index of assessed guidelines is shown in Table 3.

**Table 1:**
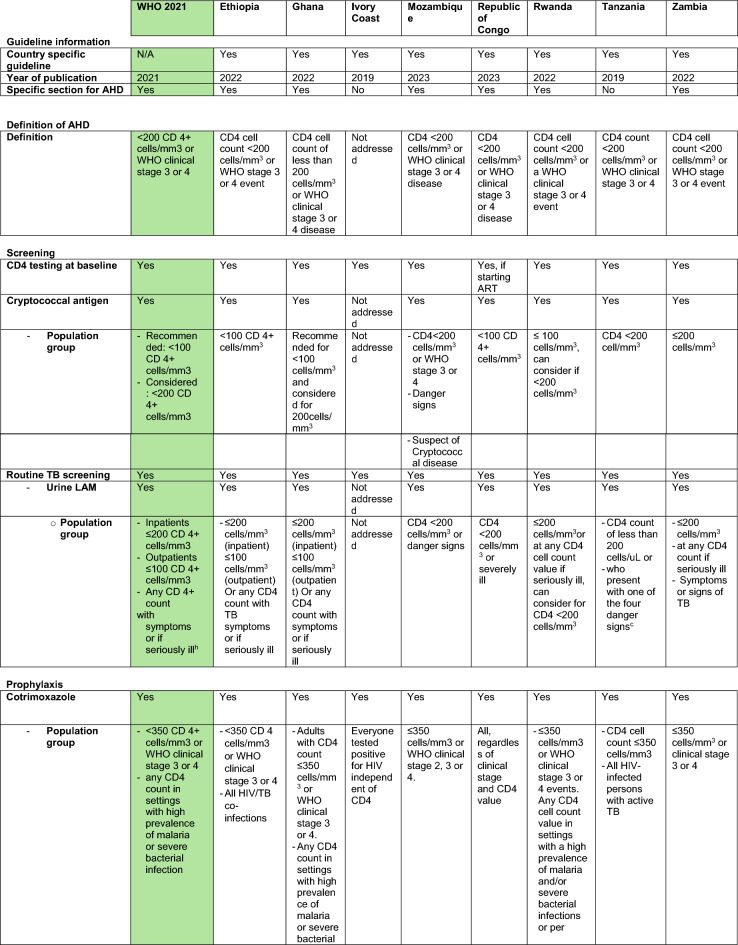

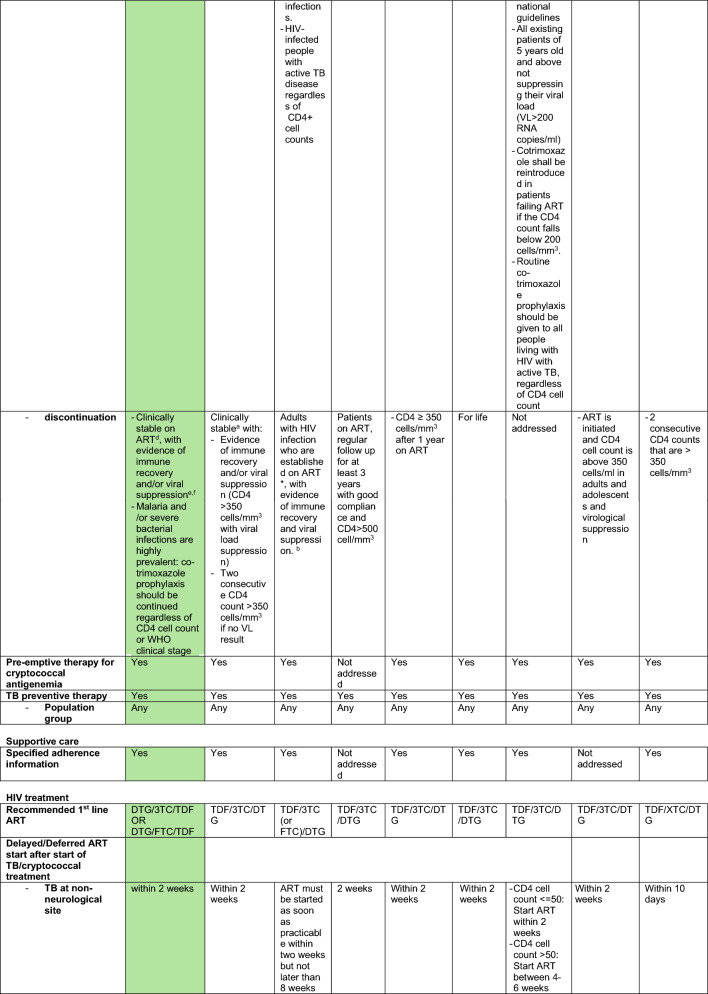

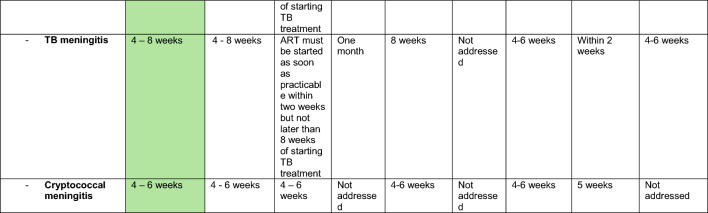
Extracted data country specific guidelines

green: reference (WHO 2021)

^a^Individuals on ART for at least one year without any new WHO clinical stage 2, 3 or 4 events

^b^The corresponding footnote in the guideline cannot be identified

^c^Respiratory rate greater than 30 breaths/minute, temperature exceeding 39 °C, heart rate more than 120 beats/minute, and/or unable to walk unaided

^d^Clinically stable adults are defined as individuals receiving ART for at least one year without any new WHO clinical stage 2, 3, or 4 events

^e^CD4 cell count > 350 cells/mm^3^, with suppression of viral loads, is considered immune recovery (some countries may adopt a threshold of CD4 cell count > 500 cells/mm^3^)

^f^WHO recognizes that in settings with low prevalence of malaria and severe bacterial infection in which co-trimoxazole is used primarily as prophylaxis for some AIDS-associated opportunistic infections (Pneumocystis jirovecii pneumonia and toxoplasmosis), guidelines exist for adults living with HIV discontinuing co-trimoxazole when there is evidence of suppressed viral loads and immune recovery at CD4 cell count > 200 cells/mm^3^ and they have been receiving ART for at least one year

^g^“Seriously ill” is defined based on four danger signs: respiratory rate of more than 30/min, temperature of more than 39 °C heart rate of more than 120/minute and unable to walk unaided

^h^Respiratory rate ≥ 30 breaths per minute; heart rate ≥ 120 beats per minute; or unable to walk unaided

*3TC* Lamivudine

*AHD* advanced HIV Disease

*ART* anti retroviral therapy

*DTG* Dolutegravir

*FTC* Emtricitabine

*LAM* lipoarabinomannan

*TDF* Tenofovir dixoproxil fumerate

**Table 2:**
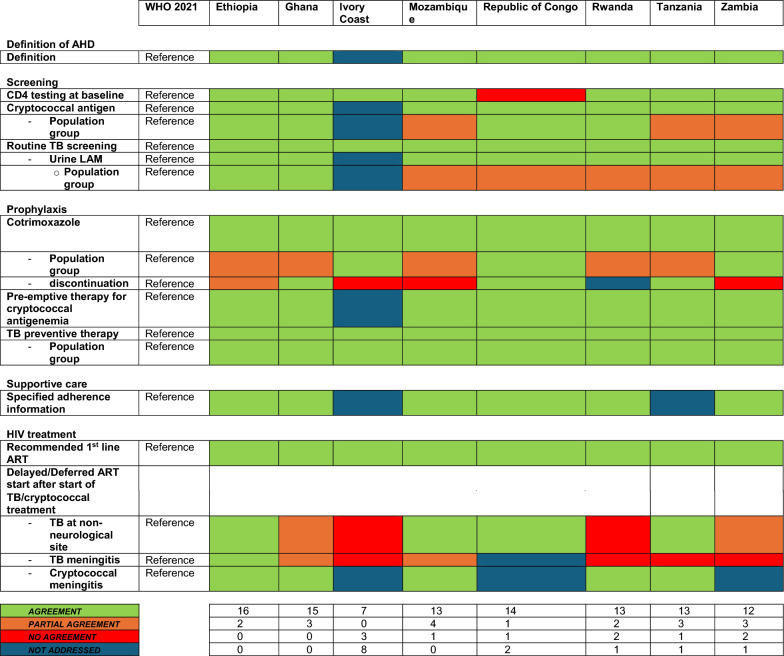
Agreement of country specific guidelines to WHO 2021

*AHD* advanced HIV Disease

*ART* anti retroviral therapy

*LAM* lipoarabinomannan

**Table 3:** Index of national guidelines

Country	Name	References
Ethiopia	National Guidelines for Comprehensive HIV Prevention, Care and Treatment	[13]
Ghana	Consolidated Guidelines for HIV Care in Ghana	[14]
Ivory Coast	Directives 2019 de prévention et de prise en charge de personnes vivant avec le VIH en Côte D'Ivoire	[15]
Mozambique	Guião de cuidados do HIV do Adulto, Adolescente Grávida, Lactante e Criança	[16]
Republic of Congo	Lignes directrices relatives à l'utilisation de médicaments antirétroviraux pour le traitement et la prévention de l'infection à VIH au Congo	[17]
Rwanda	Guidelines for HIV Prevention, Treatment and Care in Rwanda	[18]
Tanzania	National Guidelines for the Management of HIV and AIDS	[19]
Zambia	Zambia Consolidated Guidelines for Treatment and Prevention of HIV Infection	[20]

### Definition of AHD

The Ivory Coast guideline did not include a definition of AHD. All other national guidelines employed the same definition as the WHO, considering a CD4 + cell count < 200 cells/mm^3^ or a WHO clinical stage 3 or 4 disease as AHD.

### Screening

#### CD4 testing

All national guidelines recommended CD4 count testing for people with HIV (PWH) entering or re-entering antiretroviral therapy (ART) care, which is in line with the WHO 2021 recommendations. However, the Republic of Congo recommended performing CD4 testing only for those in whom ART is started.

#### Cryptococcal disease

Except of Ivory Coast, all guidelines recommended serum cryptococcal antigen testing, but there were differences in the population to be tested, resulting in partial agreement for the documents of Mozambique, Tanzania, and Zambia. Based on the WHO guideline (recommended < 100 cells/mm^3^, considered: < 200 CD4 + cells/mm^3^) we counted < 100 cells/mm^3^ as agreement and < 200 CD4 + cells/mm^3^ as partial agreement. Tanzania and Zambia guidelines recommended testing for CD4 +  < / ≤ 200 cells/mm^3^. The Mozambique guidelines recommended testing if additional criteria are met, including danger signs or suspicion of cryptococcal disease, or WHO stage 3 or 4 disease.

#### Tuberculosis

In line with the WHO, all national guidelines recommended screening for TB. The use of urine lipoarabinomannan (LAM) testing to screen for tuberculosis was discussed in all national guidelines except those from Ivory Coast. Only the Ghanaian and Ethiopian guidelines separated the targeted population by in- and out-patient as per WHO 2021 guidance (inpatient: ≤ 200 cells/mm^3^, outpatient ≤ 100 cells/mm^3^, or any CD4 count with symptoms or if seriously ill). All guidelines recommended the use of urine LAM in patients who are severely ill or have danger signs, independently of CD4 count.

### Prophylaxis for opportunistic infections

#### Cotrimoxazole

All national guidelines recommended cotrimoxazole (CTX) prophylaxis but there were differences in the criteria for timing of initiation and discontinuation. According to the WHO 2021 guidance, CTX should be provided to all PHW with a CD4 cell count of less than 350 cells/mm^3^ or clinical stage 3 or 4 disease, or in settings with high prevalence of malaria or severe bacterial infection. The Zambian guidelines recommended starting CTX if the CD4 counts is < 350 cells/mm^3^ or with clinical stage 3 or 4 disease. Ivory Coast and Republic of Congo guidelines recommended starting CTX in all PWH irrespective of the CD4 count, based on the high prevalence of malaria or severe bacterial infections. Mozambique guidelines expanded the use of CTX to also include patients with WHO clinical stage 2. Ethiopia, Ghana, Tanzania, and Rwanda guidelines recommended CTX for all PWH with active tuberculosis regardless the level of CD4, with differences in other indications. Except the use of CTX in patients with tuberculosis, guidelines of Ghana recommended CTX in line with the WHO 2021; Rwanda guidelines considered virologic failure (HIV RNA > 200 copies/ml) as a criterion for treatment; Ethiopian and Tanzania guidelines did not provide additional clinical criteria for initiating CTX prophylaxis.

All guidelines except those from Rwanda provided guidance for discontinuation of CTX for PWH. The WHO 2021 stated that CTX can be stopped if a patient is stable on ART with immune recovery or viral suppression. Guidelines from Ghana, and Tanzania aligned with the WHO guidance. In settings with a high burden of malaria or severe bacterial infections, CTX should be continued regardless of CD4 count or clinical stage, which is recommended in the guidelines of the Republic of Congo. Ethiopian guidelines include an option to discontinue prophylaxis with combining HIV viral load testing and CD4 testing. All other national guidelines recommended discontinuing CTX prophylaxis based on CD4 counts.

#### Cryptococcal antigenemia and tuberculosis preventive therapy

Other than the guideline of Ivory Coast, which do not address this item, all guidelines recommended pre-emptive therapy for cryptococcal antigenemia. All national guidelines recommended tuberculosis preventive therapy for all PWH.

### Supportive care interventions

The Guidelines of Ivory Coast and Tanzania did not provide any information about supportive care interventions. Recommendations for adherence for people with AHD from the other countries included measures such as home visits or specific communications interventions.

### Antiretroviral therapy

#### Regimen

The recommended first-line ART regimen for all countries was in line with the WHO guideline: Dolutegravir (DTG)/ lamivudine (3TC)/ tenofovir disoproxil fumarate (TDF) or DTG/emtricitabine (FTC)/TDF.

#### Timing of ART initiation in the presence of opportunistic infections

##### Non-neurological TB

The WHO guidelines recommend starting ART within 2 weeks for PWH with tuberculosis at a non-neurological site, independent of CD4 cell count. Guidelines from Ethiopia, the Republic of Congo, Mozambique, and Tanzania provided the same recommendation; Zambian guidelines recommend a start within ten days. Ivory Coast guidelines recommended deferring ART initiation for 2 weeks. Rwandan guidelines took a similar approach but suggested starting ART within 4–6 weeks for CD4 counts above 50 cells/mm^3^. Ghana guidelines recommended to start ART as soon as possible and within two weeks if practicable but latest after 8 weeks and initiation should be deferred if clinical symptoms suggest meningitis.

##### Tuberculous meningitis (TBM)

WHO 2021 guidelines recommend delaying start of ART for 4–8 weeks for patients with TBM. Ethiopian guidelines provided the same information. Guidelines from Ghana, and Mozambique recommended slightly different timings for ART initiation, but were similar to WHO recommendations. The guideline of Ivory Coast would start as early as 4 weeks, Rwanda and Zambia within 4–6 weeks. The guidelines of the Republic of Congo did not specify any timing. Tanzania guideline stated to start ART as soon as possible and within 2 weeks after TB treatment.

##### Cryptococcal meningitis

Guidelines from Ethiopia, Ghana, Mozambique, and Rwanda recommended deferring start of ART for 4–6 weeks for people with cryptococcal disease, which is consistent with the WHO 2021 guidelines. Tanzania’s guideline recommended deferral for 5 weeks (considered as agreement). The Zambian guideline recommended deferral of ART but do not specify any time period. The Republic of Congo guideline did not provide a clear timeline and the guideline from Ivory Coast did not address this issue.

### Overall agreement

**Fig. 1:**
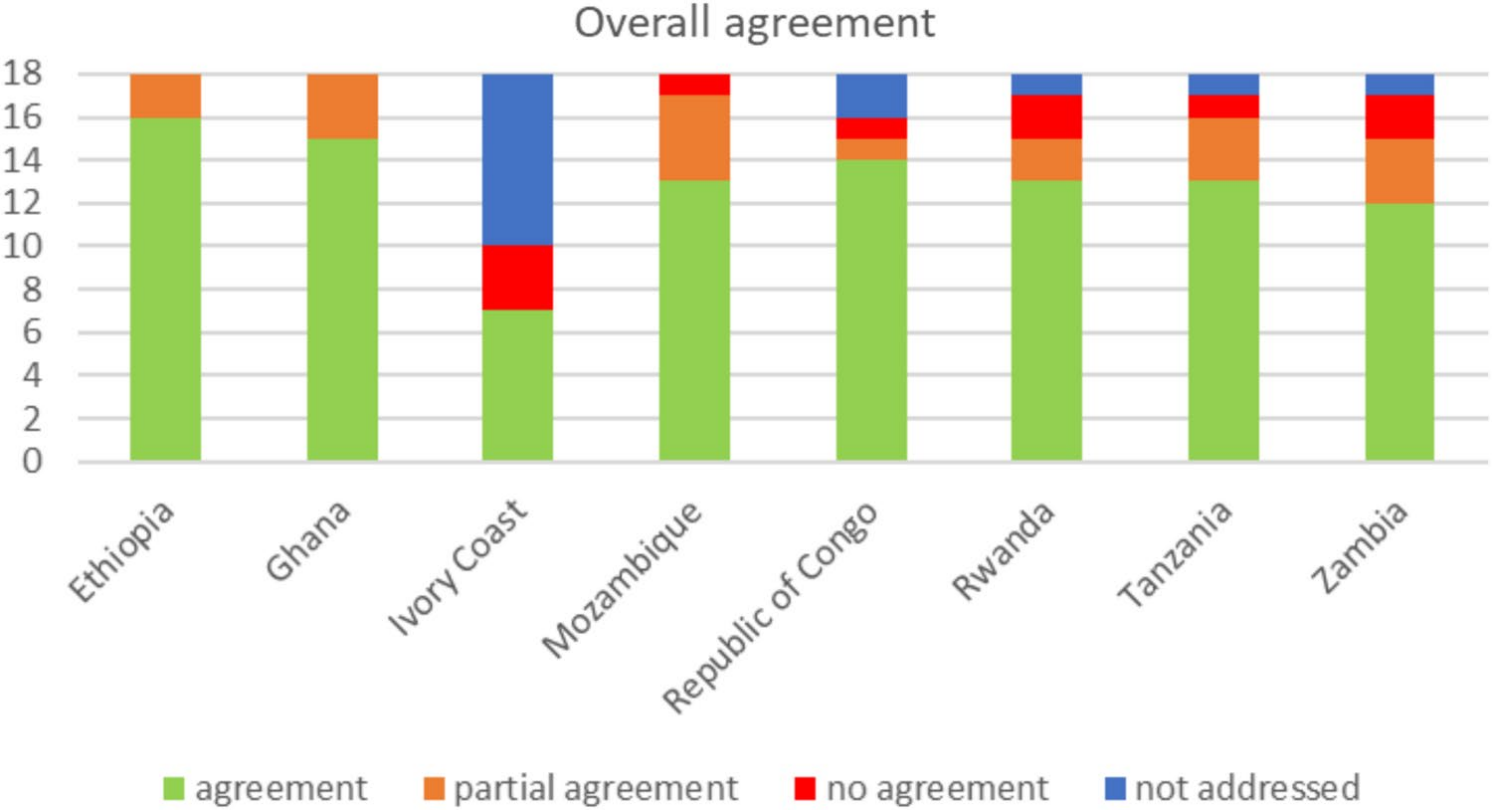
Overall agreement by country

**Fig. 2:**
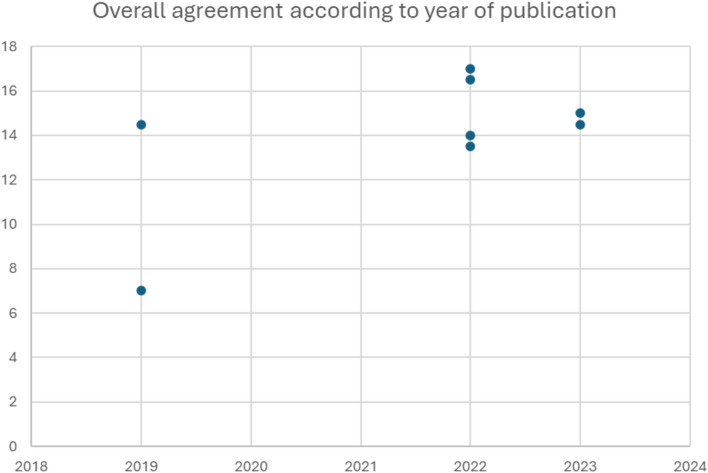
Overall agreement according to year of publication

Overall agreement for all assessed items (total 18) ranged from 7.0 for the Ivory Coast guideline to 17.0 for the guidelines from Ethiopia (Fig. 1). None of the national guidelines had more than three items not in agreement with the WHO guidelines, and the maximum number of items not addressed by any one guideline was eight. Except the guideline of Ivory Coast (overall agreement: 7.0), all documents showed similar overall agreement (range: 13.5–17.0), irrespectively of publication date (Fig. 2).

## Discussion

We evaluated the agreement between the national guidelines of eight sub-Saharan countries participating in the REVIVE trial with the latest WHO guidelines for the management of AHD. Disagreements were minor and mostly due to not covering guideline items. Those deviating from WHO recommendations were confined to recommendations for the target patient population for urine LAM testing, the timing of initiation and discontinuation of cotrimoxazole prophylaxis and the start of antiretroviral therapy in presence of tuberculosis. Except for Ivory Coast, all guidelines addressed most items included in the WHO guidance, highlighting the comprehensive nature of the national AHD guidelines. Compared with our previous report that evaluated the six vanguard countries participating in REVIVE, the current review shows less disagreement for the different indications for deferred ART [12].

All 14 countries participating in the REVIVE trial (six in the previous and eight in the current review) have their own national guideline, and any substantive differences from the WHO guidelines appear to reflect country-specific considerations, such as burden of malaria for CTX prophylaxis or available resources. The importance of local context to implement the WHO guidelines is highlighted in the WHO 2017 document [2].

All national guidelines were published after the first WHO document addressing AHD management in 2017 [2], and only the Tanzania and Ivory Coast guidelines were published before the comparator for this review (WHO 2021) [12]. However, all guidelines–except Ivory Coast–showed high agreement, irrespectively of the publication date. This is in contrast to our previous report, indicating a trend towards a higher agreement score over time.

The first step of managing AHD is to identify the patient population based on universally accepted definition. Correlation of CD4 count < 200 cells/mm^3^ with the WHO clinical stage criteria for advanced HIV (stage 3 and 4) is poor [21–23]. For instance, people with a CD4 count of < 100 cells/mm^3^ may present without stage 3 or 4 disease, and therefore reliance on clinical staging alone may fail to identify those at highest risk [7]. All national guidelines recommended CD4 testing for PWH entering or re-entering care, as this enables clinicians to identify AHD even in asymptomatic individuals. If CD4 testing is not available, some guidelines emphasized the use of WHO clinical stage assessment. A recently published WHO policy brief addressing the management of opportunistic infections in AHD supports CD4 testing to detect AHD and determine eligibility for CTX prophylaxis [24]. Ehrenkranz *and colleagues* highlight the importance of CD4 testing for risk stratification, in the context of declining use of routine testing based on the perception by funders that routine testing has limited cost effectiveness [25]. As a consequence of reduced donor and governmental funding support for CD4 testing, some manufacturers are withdrawing quantitative CD4 testing devices from the market [26].

Based on the WHO clinical stage classification, people with pulmonary tuberculosis or extra-pulmonary tuberculosis are defined as having stage 3 or 4 disease and are therefore considered to have AHD [27]. The WHO guideline recommends starting cotrimoxazole in all patients with a CD4 cell count of < 350 cells/mm^3^ or clinical stage 3 or 4 disease. The national guidelines of three countries separately recommend the use of CTX in people with active tuberculosis; this is likely based on a randomised trial performed in the Ivory Coast before ART was widely available showing reduction in mortality and hospital admission with this approach [28].

In our previous report we found that none of the six national guidelines provided different CD4 cut offs for Urine LAM testing for inpatients and outpatients [11]. In the current report, two national guidelines provided such guidance.

According to WHO, ART should be started within seven days of HIV diagnosis except for those also diagnosed with cryptococcal meningitis or tuberculous meningitis [12]. Both these infections are important drivers of mortality in PWH [8, 29], and immediate ART initiation is associated with harm [12, 30]. Some national guidelines still retain specific recommendations for ART timing among people with non-neurological TB, based on CD4 thresholds. Only one guideline was in exact agreement with the WHO 2021 guideline regarding the deferral of ART in presence of TB meningitis.

In summary we found that national guidelines for the management of AHD from eight countries in sub-Saharan Africa with a total population of more than 300 million people have a high level of agreement with the most recently updated 2021 WHO guidelines.

## Data Availability

Data is provided within the manuscript.

## Abbreviations

3TC: Lamivudine
AHD: Advanced HIV disease
ART: Antiretroviral therapy
CTX: Cotrimoxazole
CD4: Cluster of differentiation 4
DTG: Dolutegravir
FTC: Emtricitabine
HIV: Human immunodeficiency virus
LAM: Lipoarabinomannan
PWH: People living with HIV
TB: Tuberculosis
TBM: Tuberculous meningitis
TDF: Tenofovir disoproxil fumarate
WHO: World Health Organization
